# Editorial: Advances in Parasitic Weed Research

**DOI:** 10.3389/fpls.2018.00236

**Published:** 2018-03-07

**Authors:** Diego Rubiales, Mónica Fernández-Aparicio, Maurizio Vurro, Hanan Eizenberg

**Affiliations:** ^1^Institute for Sustainable Agriculture, CSIC, Córdoba, Spain; ^2^Agroécologie, AgroSup Dijon, INRA, Université Bourgogne Franche-Comté, Dijon, France; ^3^Institute of Sciences of Food Production, CNR, Bari, Italy; ^4^Agricultural Research Organization, Newe Ya'ar Research Center, Ramat Yishay, Israel

**Keywords:** striga, parasitic weeds, broomrape, weed management, resistance

Over 4,000 plant species parasitize other plants to obtain water and nutrients. A few of these species have become weedy posing a tremendous threat to agriculture. The most damaging to annual crops are the root parasitic weeds, particularly broomrapes (*Orobanche* and *Phelipanche* spp.) and witchweeds (*Striga* spp.), which are extended over large agricultural areas in Europe, Africa and Asia. A problem of less magnitude but of increasing importance is inflicted by the parasitic weeds *Alectra, Aeginetia, Buchnera*, and *Rhamphicarpa*. To date, advances in control strategies have concentrated on agronomic practices, resistant varieties and the use of herbicides, often showing limited level of control particularly in low-input crops. Novel control programmes should be sympathetic to agricultural extensification while exerting minimal harmful effects on the environment. In addition, global environmental changes, together with changing land use patterns with less dependency on synthetic herbicides is favoring the diffusion of parasitic weeds to new geographical areas and farming systems. Thus, besides control methods, it is imperative to prevent the spread of parasitic weeds and to impose, where possible quarantine regulations.

The goal of this Research Topic was to present new research dealing with advanced management of parasitic weeds, but also new knowledge on its mechanism, the biology and physiology of the processes of parasitic weed germination and crop infection, their genetics and population dynamics, and to present novel sources of crop resistance, in order to offer new understanding of these enigmatic plants and their better management. Here a brief outline of its contents, briefly describing the articles grouped by subjects.

## Detection and management

The seed bank of root parasitic weeds rapidly develops as enormous amounts of seeds are produced and spread in the field. Cohen et al. used ecoinformatics and spatial analysis to study the spread patterns of *Phelipanche aegyptiaca* in processing tomatoes. To limit parasitic weed spread, drastic inspection measures are needed to identify seed contaminations of commercial seed lots. In order to facilitate the identification of most noxious broomrape species from a single seed Rolland et al. developed a High Resolution Melting assay based on plastidial genes amplification.

Early detection of underground parasites is essential for an efficient control and with this objective Cochavi et al. showed the potential of hyperspectral tools to detect early underground stages of *Orobanche cumana* parasitism on sunflower roots. Findings emphasized the correlation between specific reflectance changes in the visible to shortwave infrared range and levels of various nutrients in sunflower plants. In addition, Ortiz-Bustos et al. investigated the potential of UV-induced multicolor fluorescence imaging in the red and far-red spectral regions for an early detection of *O. cumana* infection in sunflower. This technique might have a particular interest for early phenotyping in sunflower breeding programs.

Models to predict the underground parasite development are a valuable tool for herbicide applications. Pérez-de-Luque et al. suggested a model based on thermal time and correlating growing degree-days with the different developmental stages of *Orobanche crenata* in three legume crops. Development of broomrape was highly temperature-related, with thermal time appearing as a valuable tool for describing the parasite growth and establishing the developmental stage of the infection. Similarly, Cochavi et al. developed a thermal time model to predict the effect of *P. aegyptiaca* parasitism dynamics on carrot growth. Two different nonlinear models were developed for optimal prediction of *P. aegyptiaca* parasitism dynamics.

The knowledge of competitive relations between parasitic weeds and their host crops can allow determining the intervention thresholds of control measurements in integrated pest management programs. Fernández-Aparicio et al. modeled productivity losses caused by *O. crenata* infection in three legume crops showing trends in dry matter allocation in relation to infection severity. The increase of resources allocated within the parasite was concomitant to reduction of host seed yield indicating that parasite growth and host reproduction compete directly for resources within a host plant. In addition, Moreau et al. quantified the intensity by which *Phelipanche ramosa* plants draw assimilates from their host and analyzed whether it varied with host species, phenological stage and growth rate. Results will be incorporated into a mechanistic model in order to analyze the effect of parasitic plant species on weed community assembly and to design new cropping systems for controlling *P. ramosa*.

Most of the parasitic weed damage in the crop occurs during early stages of their underground infection, and by the time the parasite is visible emerged from the soil surface, any control method is useless as the damage to the crop has already been done. Fernández-Aparicio et al. reviewed the underground biology of broomrape weeds and the control strategies to inhibit their parasitism. The vulnerability of some underground events key for the intractable parasitism, such as crop-induced seed germination or haustorial development were reviewed as inhibition targets for plant protection programs. Root parasitic seed germination is triggered by host-derived signals upon which it invades the host root, and strigolactones are the main germination stimulants. Cheng et al. studied the role of endogenous strigolactones and their interaction with ABA during the infection of tomato by *P. ramosa*. These findings may have implications for future control strategies, including improvement of parasitic plant resistance.

Under farming conditions, broomrape control is difficult and in most cases, only amino acid biosynthesis-inhibiting herbicides allow an acceptable control level. Dor et al. studied the mode of action of the aromatic and branched-chain amino acid biosynthesis-inhibiting herbicides imazapic and glyphosate in broomrapes, and showed that both herbicides inhibited *P. aegyptiaca* callus growth and altered the free amino acid content. Both EPSPS and ALS are active in *P. aegyptiaca* callus and flowering shoots and are inhibited by glyphosate and imazapic, respectively. In a further study Shilo et al. showed that glyphosate mechanism of action is probably more complex, and it probably have a secondary effect in *P. aegyptiaca* as a consequence of its primary target inhibition, via inhibition of the translocation of phloem-mobile solutes to the parasite. As an alternative to synthetic herbicides, certain amino acids can induce similar effects of amino acid-inhibitory herbicides in plant growth due to feedback inhibition of metabolic pathways, and those inhibiting the growth of parasitic weeds have the potential for parasitic weed management. Nzioki et al. developed amino acid-overproducing lines of *Fusarium oxysporum* f.sp. *strigae* and validated its enhanced biocontrol action during management of *Striga hermonthica* on smallholder farms. Integration of this plant pathogen with enhanced virulence to *Striga* management in maize can significantly increase maize yield of smallholder farmers in Kenya. In addition, the orobanchicidal effect of direct application of amino acids to the soil was proved effective in field by Fernández-Aparicio et al. inhibiting *Orobanche minor* parasitism on red clover.

Alternative control systems were developed in tomato by Eizenberg et al. which showed that the addition of biochar to the pot soil resulted in inhibition of *P. aegyptiaca* infection. Biochar did not affect the tomato exudation of stimulants of parasitic germination, nor changed the penetration ability of the parasite. The major cause for the decrease in germination percentage was physical adsorption of the stimulant molecule by the biochar. In addition, Venezian et al. studied the influence of plant growth regulators on *P. aegyptiaca* development and its control efficacy showing that maleic hydrazide is a potent inhibitor of the early stages of parasitism, namely attachment and the tubercle stage.

## Host-parasite interactions

Parasitic plants connect to the host phloem through the haustorium and act as supernumerary sinks for the host-derived photoassimilates, primarily sucrose. Péron et al. showed direct phloem connections at the host-parasite interface and evidenced the dominant apoplastic pathway for phloem unloading in major vegetative sinks of the parasite and suggesting the pivotal role of sucrose transporters in sucrose unloading in *P. ramosa* sinks. The complex endophytic structure formed by parasitic plant species often represents a challenge in the study of the host-parasite interface. Teixeira-Costa and Ceccantini compared the infestation pattern of two mistletoe species by High Resolution X-ray Computed Tomography. The combination of three-dimensional models of the infestation with anatomical analysis provided a broader understanding of the host-parasite connection.

Nativ et al. studied the metabolic profile of *P. aegyptiaca* during its developmental stages, by using GC-MS analysis. The first three developmental stages had diverse metabolomics profiles, but the latter two did not differ from the mature stage. Compared to other organs, floral buds had higher levels of free amino acids and total nitrogen, whereas flowers accumulated higher levels of simple sugars such as sucrose, and the putative precursors for nectar synthesis, color, and volatiles. This suggests that the reproductive organs have the ability to accumulate metabolites that are required for the production of seeds and as a source of energy for the reproductive processes.

There is considerable controversy on the taxonomy of the Orobanchaceae. Tóth et al. studied floral volatile organic compounds as an additional tool for species identification, drafting a blend-based phylogeny that supported known taxonomic groups and helped to clarify the uncertainty in some taxonomical groups.

Broomrapes establish direct connections with the host vascular system. This connection allows the exchange of endophytic microorganisms inhabiting the tissues of both plants. Kruh et al. characterized the endophytic composition in *P. aegyptiaca* during the parasitization process, evaluating the eventual corresponding changes in the host root. A *Pseudomonas* sp. strain hosted by tomato roots suppressed approximately 80% *P. aegyptiaca* seed germination and significantly reduced *P. aegyptiaca* parasitism, highlighting the potential of alternative environmentally friendly approaches for parasitic weed control.

## Resistance and biotechnology

Under the prevailing farming conditions in sub-Saharan Africa, the use of resistant crop varieties has been proposed as a cost-effective and easy-to-adopt strategy for *Striga* management. Mbuvi et al. showed that wild sorghum accessions are an important reservoir of *Striga* resistance that could be used to expand the genetic basis of cultivated sorghum for resistance to the parasite. Samejima et al. reported and characterized resistance to *S. hermonthica* in upland rice.

Only moderately broomrape resistant faba bean cultivars are available to farmers. Rubiales et al. dissected resistance components in faba bean accessions against a number of infective and non-infective broomrape species. The intermediate levels of resistance of cv. Baraca acted against all broomrape populations and species studied, confirming previous reports on the stability of its resistance, whose identified mechanisms were due to (1) low induction of seed germination; (2) negative tropism of germinated seeds with radicles growing away from faba bean roots; (3) necrosis of radicles that had successfully contacted faba bean roots; (4) necrosis of formed broomrape tubercles.

Breeding sunflower for resistance to *O. cumana* is challenging as races have evolved. The parasitic interaction between sunflower and *O. cumana* generally follows a gene-for-gene model, with resistance in sunflower and avirulence in *O. cumana* controlled by dominant alleles at single loci. Race F spread in the middle race, named “F” spread in the middle 90's and was contained thanks to resistant sunflower hybrids. However, also this resistance has been recently broken down by race G. Martín-Sanz et al. reports how increased virulence in Spanish populations is associated with greater genetic diversity. They distinguished race G_GV_ from other race G populations and suggested that increased virulence was not caused by new introductions but might have resulted from admixture of populations followed by recombination of avirulence genes. Because of this frequent development of more virulent races, attention is being paid to quantitative resistances. Louarn et al. reported that resistance introduced from *Helianthus debilis* is controlled by specific QTLs for different parasitism stages. Different QTLs were identified for each race (F from Spain and G from Turkey) and for the three stages of broomrape development. The results indicate that there are several quantitative resistance mechanisms controlling the infection by *O. cumana* that can be used in sunflower breeding.

Gene-silencing is a powerful technology for RNA degradation in plants. Dubey et al. suggested an improved strategy for the control of parasitic weeds based on the simultaneous trans-specific gene-silencing of three parasite genes. Two strategies were used to express dsRNA containing selected sequences of *P. aegyptiaca* genes *PaACS, PaM6PR*, and *PaPrx1*. The results showed the movement of mobile exogenous siRNA from the host to the parasite, leading to the impaired expression of essential parasite target genes.

## Author contributions

All authors listed have made a substantial, direct and intellectual contribution to the work, and approved it for publication.

### Conflict of interest statement

The authors declare that the research was conducted in the absence of any commercial or financial relationships that could be construed as a potential conflict of interest. The reviewer RA declared a past co-authorship with one of the authors HE to the handling Editor.

